# Poly[[(μ_2_-benzene-1,4-dicarboxyl­ato-κ^4^ *O* ^1^,*O* ^1′^:*O* ^4^,*O* ^4′^)(μ_2_-di-4-pyridyldiazene-κ^2^ *N* ^1^:*N* ^1′^)cobalt(II)] *N*,*N*-dimethyl­formamide disolvate hemihydrate]

**DOI:** 10.1107/S1600536809030189

**Published:** 2009-08-08

**Authors:** Chao-Xia Chu, Ying Zhang, Hu Zhou, Ai-Hua Yuan

**Affiliations:** a School of Material Science and Engineering, Jiangsu University of Science and Technology, Zhenjiang 212003, People’s Republic of China

## Abstract

In the title compound, {[Co(C_8_H_4_O_4_)(C_10_H_8_N_4_)]·2C_3_H_7_NO·0.5H_2_O}_*n*_, the Co^II^ atom is six-coordinated by four O atoms from two benzene-1,4-dicarboxyl­ate (H_2_bdc^2−^) groups and two N atoms from two 4,4′-azopyridine (4,4′-azpy, or di-4-pyridyldiazene) ligands, leading to a distorted octa­hedral geometry. The structure consists of two-dimensional corrugated sheets with a 4^4^ topology in an …*ABAB*… packing pattern stacking along the *a* axis. The separation of the adjacent corrugated sheets is *ca*. 8.561 (2)  Å (Co⋯Co distance) along the *a* axis. The uncoordinated water molecule is half-occupied. The crystal structure is stabilized by O—H⋯N and C—H⋯O hydrogen-bonding inter­actions.

## Related literature

For background to metal-organic framework (MOF) materials, see: Halder & Kepert (2002 ▶); Murray & Cashion (2002 ▶); Rosi *et al.* (2003 ▶); Rowsell *et al.* (2005 ▶); Seo *et al.* (2000 ▶). For compounds containing H_2_bdc or 4,4′-azpy ligands, see: Halder *et al.* (2005 ▶); Jia (2007 ▶). 
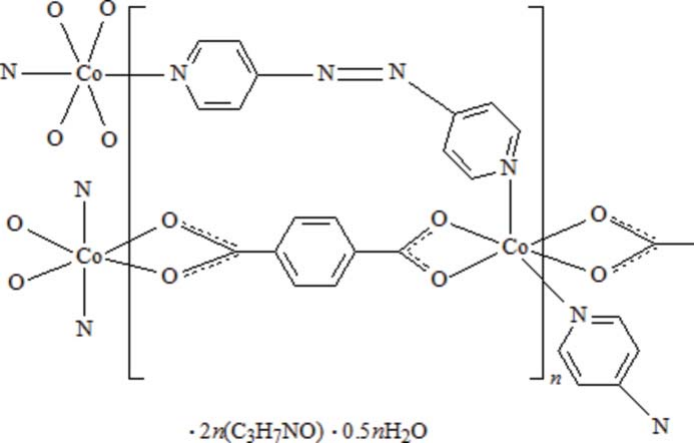


## Experimental

### 

#### Crystal data

[Co(C_8_H_4_O_4_)(C_10_H_8_N_4_)]·2C_3_H_7_NO·0.5H_2_O
*M* *_r_* = 562.45
Orthorhombic, 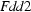

*a* = 32.441 (4) Å
*b* = 34.138 (4) Å
*c* = 10.1972 (12) Å
*V* = 11293 (2) Å^3^
*Z* = 16
Mo *K*α radiation
μ = 0.66 mm^−1^
*T* = 291 K
0.25 × 0.20 × 0.08 mm

#### Data collection

Bruker SMART APEX CCD diffractometer
Absorption correction: multi-scan (*SADABS*; Bruker, 2004 ▶) *T*_min_ = 0.84, *T*_max_ = 0.88 (expected range = 0.906–0.949)
21948 measured reflections
5500 independent reflections
4262 reflections with *I* > 2σ(*I*)
*R*_int_ = 0.062

#### Refinement

*R*[*F* ^2^ > 2σ(*F* ^2^)] = 0.057
*wR*(*F* ^2^) = 0.134
*S* = 1.07
5500 reflections
335 parameters
1 restraint
H-atom parameters constrained
Δρ_max_ = 0.35 e Å^−3^
Δρ_min_ = −0.36 e Å^−3^
Absolute structure: Flack (1983 ▶), 2564 Friedel pairs
Flack parameter: 0.04 (2)

### 

Data collection: *SMART* (Bruker, 2004 ▶); cell refinement: *SAINT* (Bruker, 2004 ▶); data reduction: *SAINT*; program(s) used to solve structure: *SHELXS97* (Sheldrick, 2008 ▶); program(s) used to refine structure: *SHELXL97* (Sheldrick, 2008 ▶); molecular graphics: *SHELXTL* (Sheldrick, 2008 ▶) and *DIAMOND* (Brandenburg, 2006 ▶); software used to prepare material for publication: *SHELXL97*.

## Supplementary Material

Crystal structure: contains datablocks I, global. DOI: 10.1107/S1600536809030189/at2838sup1.cif

Structure factors: contains datablocks I. DOI: 10.1107/S1600536809030189/at2838Isup2.hkl

Additional supplementary materials:  crystallographic information; 3D view; checkCIF report

## Figures and Tables

**Table 1:** Selected geometric parameters (Å, °)

N1—Co1	2.079 (4)
N4—Co1^i^	2.062 (4)
O1—Co1	2.153 (3)
O2—Co1	2.153 (3)
O3—Co1^iv^	2.059 (3)
O4—Co1^iv^	2.353 (3)

Symmetry codes: (i) 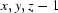; (ii) 
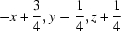
; (iii) 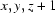; (iv) 
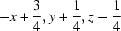
.

**Table 2:** Hydrogen-bond geometry (Å, °)

*D*—H⋯*A*	*D*—H	H⋯*A*	*D*⋯*A*	*D*—H⋯*A*
O7—H7*A*⋯N5^ii^	0.85	2.32	2.932 (7)	129
C7—H7⋯O7	0.93	2.30	3.126 (7)	147
C16—H16⋯O3	0.93	2.48	2.791 (4)	100
C17—H17⋯O1	0.93	2.49	2.800 (4)	100
C19—H19*C*⋯O1^iv^	0.96	2.37	3.150 (6)	138
C23—H23*A*⋯O6^v^	0.96	1.71	2.624 (5)	158

Symmetry codes: (ii) 
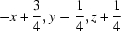
; (iv) 
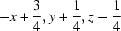
; (v) 
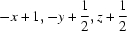
.

**Fig. 1.**
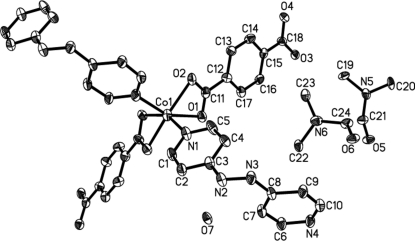
ORTEP diagram of the title compound. Displacement ellipsoids are drawn at the 30% probablity level. Hydrogen atoms are omitted for clarity.

**Fig. 2.**
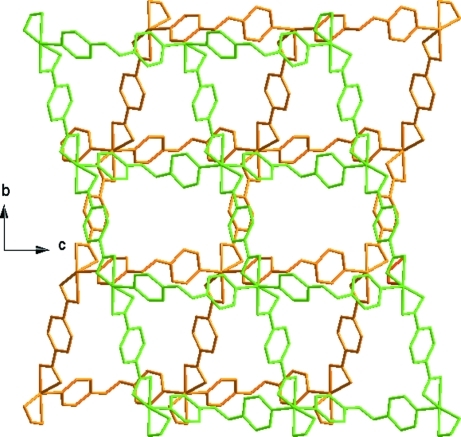
View of the stacking without interpenetration of sheets along the a axis. Hydrogen atoms, solvent DMF molecules and water molecules are not involved for clarity.

## supplementary crystallographic information

### Comment

Metal organic framework (MOF) materials have attracted much attention due to their potential functionalities such as gas storage (Rosi *et al.*, 2003; Rowsell *et al.*, 2005), sensing (Halder *et al.*, 2002), and catalysis (Seo *et al.*, 2000). The organic ligands, especially, benzene-1,4-dicarboxylate (H_2_bdc) and 4,4'-azopyridine (4,4'-azpy), play a great role on constructing the topological architectures of MOFs (Jia, 2007; Halder *et al.*, 2005). Here we employed H_2_bdc and 4,4'-azpy as mixed ligands to bridge the Co^II^ atom, obtaining the title compound by solvothermal synthesis.

In the structure of the title compound, each Co^II^ atom, lying on an inversion center, is coordinated by four oxygen atoms from two H_2_BDC groups and two nitrogen atoms from two 4,4'-azpy ligands, exhibiting a slightly distorted octahedral geometry (Fig. 1). The bond lengths of Co—O range from 2.059 (3) to 2.353 (3) Å, while the ones of Co—N are 2.079 (4) Å for Co1—N1 and 2.062 (4) Å for Co1—N4, respectively (Table 1). The Co^II^ centers are linked by H_2_bdc groups into one-dimensional infinite zigzag chains along the *b* axis in the *bc* plane. Then, the chains are further linked by 4,4'- azpy ligands along the *c* axis, resulting in two-dimensional corrugated sheets with 4^4^ topology. These corrugated sheets without interpenetration are stacking along the *a* axis in an ABAB packing mode (Fig. 2). The torsion angle of the adjacent sheets is *ca*. 45 ° in the *bc* plane, while the separation between adjacent corrugated sheets is *ca*. 8.56 Å (Co···Co distance) along the *a* axis.

The crystal structure is stabilized by O—H···N and C—H···O hydrogen bonding interactions (Table 2).

### Experimental

A mixture of CoCl_2_.6H_2_O (23.8 mg, 0.1 mmol), H_2_bdc (16.6 mg, 0.1 mmol), 4,4'-azpy (18.4 mg, 0.1 mmol) and DMF (*N*, *N*-dimethylformamide) (10 ml) was stirred for 15 min at room temperature and then transferred into a Teflon-lined stainless-steel vessel. The mixture was heated at 433 K for two days under autogenous pressure. After cooling the resulting solution to room temperature with the rate of 10 °C/h, purple and layer-shaped crystals were obtained. Analysis calculated for Co_2_N_12_O_13_C_48_H_54_: C 51.25, H 4.80, N 14.93%; found: C 51.16, H 4.65, N 14.92%.

### Refinement

The C(H) atoms of the H_2_bdc ligands, 4,4'-azpy ligands, and solvent DMF molecules were all placed in calculated position [C—H = 0.93 Å or 0.96 Å] and refined using a riding model, with *U*_ĩso_(H) = 1.2*U*_eq_(C) or *U*_ĩso_(H) = 1.5*U*_eq_(C). The O(H) atoms of the water molecules were located in a difference Fourier map and refined as riding [O—H = 0.85 Å], with *U*_ĩso_(H) = 1.2*U*_eq_(O).

### Crystal data

**Table:**

[Co(C_8_H_4_O_4_)(C_10_H_8_N_4_)]·2C_3_H_7_NO·0.5H_2_O	*F*(000) = 4672
*M**_r_* = 562.45	*D*_x_ = 1.323 Mg m^−^^3^
Orthorhombic, *F**d**d*2	Mo *K*α radiation, λ = 0.71073 Å
Hall symbol: F 2 -2d	Cell parameters from 3875 reflections
*a* = 32.441 (4) Å	θ = 2.2–24.4°
*b* = 34.138 (4) Å	µ = 0.66 mm^−^^1^
*c* = 10.1972 (12) Å	*T* = 291 K
*V* = 11293 (2) Å^3^	Pale, purple
*Z* = 16	0.25 × 0.20 × 0.08 mm

### Data collection

**Table:**

Bruker SMART APEX CCD diffractometer	5500 independent reflections
Radiation source: sealed tube	4262 reflections with *I* > 2σ(*I*)
graphite	*R*_int_ = 0.062
φ and ω scans	θ_max_ = 26.0°, θ_min_ = 1.7°
Absorption correction: multi-scan (*SADABS*; Bruker, 2004)	*h* = −40→40
*T*_min_ = 0.84, *T*_max_ = 0.88	*k* = −42→41
21948 measured reflections	*l* = −12→12

### Refinement

**Table:**

Refinement on *F*^2^	Secondary atom site location: difference Fourier map
Least-squares matrix: full	Hydrogen site location: inferred from neighbouring sites
*R*[*F*^2^ > 2σ(*F*^2^)] = 0.057	H-atom parameters constrained
*wR*(*F*^2^) = 0.134	*w* = 1/[σ^2^(*F*_o_^2^) + (0.07*P*)^2^ + 1.99*P*] where *P* = (*F*_o_^2^ + 2*F*_c_^2^)/3
*S* = 1.07	(Δ/σ)_max_ < 0.001
5500 reflections	Δρ_max_ = 0.35 e Å^−^^3^
335 parameters	Δρ_min_ = −0.36 e Å^−^^3^
1 restraint	Absolute structure: Flack (1983), 2564 Freidel pairs
Primary atom site location: structure-invariant direct methods	Flack parameter: 0.04 (2)

### Special details

**Table:**

Geometry. All e.s.d.'s (except the e.s.d. in the dihedral angle between two l.s. planes) are estimated using the full covariance matrix. The cell e.s.d.'s are taken into account individually in the estimation of e.s.d.'s in distances, angles and torsion angles; correlations between e.s.d.'s in cell parameters are only used when they are defined by crystal symmetry. An approximate (isotropic) treatment of cell e.s.d.'s is used for estimating e.s.d.'s involving l.s. planes.
Refinement. Refinement of *F*^2^ against ALL reflections. The weighted *R*-factor *wR* and goodness of fit *S* are based on *F*^2^, conventional *R*-factors *R* are based on *F*, with *F* set to zero for negative *F*^2^. The threshold expression of *F*^2^ > σ(*F*^2^) is used only for calculating *R*-factors(gt) *etc*. and is not relevant to the choice of reflections for refinement. *R*-factors based on *F*^2^ are statistically about twice as large as those based on *F*, and *R*- factors based on ALL data will be even larger.

### Fractional atomic coordinates and isotropic or equivalent isotropic displacement parameters (Å^2^)

**Table:**

	*x*	*y*	*z*	*U*_iso_*/*U*_eq_	Occ. (<1)
C1	0.52950 (14)	0.07179 (14)	0.9140 (5)	0.0483 (11)	
H1	0.5231	0.0517	0.9724	0.058*	
C2	0.55879 (14)	0.06426 (13)	0.8190 (5)	0.0469 (10)	
H2	0.5719	0.0401	0.8153	0.056*	
C3	0.56831 (13)	0.09346 (13)	0.7291 (5)	0.0440 (10)	
C4	0.54745 (14)	0.12918 (13)	0.7419 (5)	0.0460 (11)	
H4	0.5529	0.1498	0.6847	0.055*	
C5	0.51897 (13)	0.13334 (13)	0.8397 (6)	0.0543 (12)	
H5	0.5050	0.1571	0.8452	0.065*	
C6	0.51064 (14)	0.08224 (13)	0.3277 (5)	0.0471 (10)	
H6	0.4900	0.0634	0.3279	0.057*	
C7	0.53796 (13)	0.08165 (13)	0.4260 (5)	0.0447 (11)	
H7	0.5355	0.0632	0.4927	0.054*	
C8	0.57026 (13)	0.10881 (12)	0.4282 (4)	0.0393 (10)	
C9	0.57199 (12)	0.13488 (13)	0.3217 (5)	0.0447 (10)	
H9	0.5932	0.1531	0.3151	0.054*	
C10	0.54305 (13)	0.13299 (13)	0.2316 (4)	0.0448 (10)	
H10	0.5449	0.1507	0.1625	0.054*	
C11	0.43039 (14)	0.16957 (14)	1.0169 (4)	0.0438 (10)	
C12	0.40496 (7)	0.20614 (7)	0.9897 (3)	0.0409 (10)	
C13	0.41490 (7)	0.24214 (8)	1.0450 (3)	0.0467 (11)	
H13	0.4377	0.2444	1.0996	0.056*	
C14	0.39070 (9)	0.27481 (6)	1.0187 (3)	0.0460 (11)	
H14	0.3973	0.2989	1.0557	0.055*	
C15	0.35657 (8)	0.27148 (7)	0.9370 (3)	0.0463 (11)	
C16	0.34663 (7)	0.23548 (8)	0.8817 (3)	0.0437 (10)	
H16	0.3238	0.2333	0.8271	0.052*	
C17	0.37083 (8)	0.20281 (6)	0.9081 (3)	0.0419 (10)	
H17	0.3642	0.1787	0.8711	0.050*	
C18	0.32954 (13)	0.30541 (13)	0.9112 (4)	0.0416 (10)	
C19	0.34469 (15)	0.31385 (14)	0.5392 (4)	0.0459 (11)	
H19A	0.3659	0.2965	0.5700	0.069*	
H19B	0.3189	0.3068	0.5790	0.069*	
H19C	0.3516	0.3403	0.5623	0.069*	
C20	0.32794 (13)	0.34669 (13)	0.3270 (5)	0.0469 (11)	
H20A	0.3427	0.3492	0.2459	0.070*	
H20B	0.3336	0.3689	0.3818	0.070*	
H20C	0.2989	0.3454	0.3094	0.070*	
C21	0.35209 (14)	0.27414 (13)	0.3303 (5)	0.0465 (10)	
H21A	0.3777	0.2632	0.3487	0.056*	
C22	0.46065 (14)	0.18349 (13)	0.4854 (5)	0.0464 (11)	
H22A	0.4494	0.1745	0.4037	0.070*	
H22B	0.4432	0.1753	0.5561	0.070*	
H22C	0.4877	0.1726	0.4971	0.070*	
C23	0.44909 (13)	0.24827 (14)	0.6023 (4)	0.0464 (11)	
H23A	0.4725	0.2557	0.6543	0.070*	
H23B	0.4313	0.2317	0.6531	0.070*	
H23C	0.4343	0.2713	0.5757	0.070*	
C24	0.47026 (13)	0.24724 (14)	0.3600 (4)	0.0453 (10)	
H24A	0.4547	0.2687	0.3336	0.054*	
N1	0.50968 (12)	0.10580 (11)	0.9280 (4)	0.0501 (10)	
N2	0.59730 (11)	0.08629 (11)	0.6303 (4)	0.0464 (9)	
N3	0.59939 (11)	0.11042 (11)	0.5299 (4)	0.0464 (9)	
N4	0.51102 (12)	0.10792 (11)	0.2297 (4)	0.0477 (10)	
N5	0.34115 (11)	0.31054 (11)	0.3948 (4)	0.0462 (9)	
N6	0.46332 (12)	0.22667 (11)	0.4842 (4)	0.0485 (9)	
O1	0.41954 (9)	0.13759 (9)	0.9692 (3)	0.0463 (7)	
O2	0.46054 (10)	0.17289 (9)	1.0912 (3)	0.0490 (8)	
Co1	0.467820 (19)	0.110280 (18)	1.08164 (6)	0.04633 (17)	
O3	0.30051 (9)	0.30257 (8)	0.8275 (3)	0.0450 (7)	
O4	0.33472 (10)	0.33757 (9)	0.9713 (3)	0.0470 (7)	
O5	0.32930 (9)	0.25672 (9)	0.2523 (3)	0.0490 (8)	
O6	0.49957 (10)	0.23314 (9)	0.2912 (3)	0.0495 (8)	
O7	0.49198 (17)	0.02737 (16)	0.6249 (6)	0.0438 (15)	0.50
H7A	0.4779	0.0440	0.6677	0.053*	0.50
H7C	0.5147	0.0233	0.6643	0.053*	0.50

### Atomic displacement parameters (Å^2^)

**Table:**

	*U*^11^	*U*^22^	*U*^33^	*U*^12^	*U*^13^	*U*^23^
C1	0.055 (3)	0.041 (2)	0.049 (3)	0.003 (2)	0.011 (2)	0.018 (2)
C2	0.052 (2)	0.045 (2)	0.044 (2)	0.0129 (19)	0.004 (2)	0.011 (2)
C3	0.038 (2)	0.045 (2)	0.049 (3)	0.0034 (19)	−0.0015 (19)	0.013 (2)
C4	0.046 (2)	0.034 (2)	0.058 (3)	−0.0052 (19)	0.008 (2)	0.011 (2)
C5	0.039 (2)	0.041 (2)	0.083 (3)	0.0057 (19)	0.018 (3)	0.023 (3)
C6	0.046 (2)	0.051 (3)	0.044 (2)	−0.0026 (19)	−0.014 (2)	0.013 (2)
C7	0.041 (2)	0.044 (3)	0.049 (3)	−0.0074 (19)	−0.0096 (19)	0.0190 (19)
C8	0.034 (2)	0.037 (2)	0.047 (2)	0.0005 (17)	0.0021 (17)	0.0160 (18)
C9	0.046 (2)	0.049 (2)	0.039 (2)	−0.0168 (19)	0.004 (2)	0.016 (2)
C10	0.045 (2)	0.046 (2)	0.043 (2)	−0.002 (2)	−0.001 (2)	0.014 (2)
C11	0.043 (2)	0.049 (3)	0.039 (2)	0.0040 (19)	0.007 (2)	0.0094 (19)
C12	0.036 (2)	0.041 (2)	0.046 (2)	−0.0004 (17)	0.0028 (19)	−0.0029 (19)
C13	0.048 (2)	0.041 (2)	0.051 (3)	0.0011 (19)	−0.019 (2)	−0.0003 (19)
C14	0.042 (2)	0.052 (3)	0.044 (2)	0.011 (2)	−0.0092 (19)	−0.011 (2)
C15	0.053 (3)	0.048 (3)	0.038 (2)	0.006 (2)	−0.015 (2)	0.008 (2)
C16	0.039 (2)	0.053 (3)	0.039 (2)	0.0139 (19)	−0.0165 (18)	−0.002 (2)
C17	0.033 (2)	0.045 (2)	0.048 (3)	0.0103 (18)	0.0037 (18)	0.0088 (19)
C18	0.042 (2)	0.035 (2)	0.048 (3)	−0.0035 (17)	−0.0090 (19)	−0.0017 (19)
C19	0.050 (2)	0.047 (3)	0.040 (2)	0.011 (2)	−0.0153 (19)	−0.0161 (19)
C20	0.048 (2)	0.045 (2)	0.048 (2)	0.0133 (18)	0.025 (2)	0.024 (2)
C21	0.050 (2)	0.048 (2)	0.041 (2)	−0.0162 (19)	0.022 (2)	−0.011 (2)
C22	0.048 (2)	0.045 (2)	0.046 (2)	−0.016 (2)	−0.019 (2)	0.018 (2)
C23	0.041 (2)	0.052 (3)	0.046 (3)	−0.0109 (19)	0.0137 (19)	0.016 (2)
C24	0.044 (2)	0.047 (2)	0.045 (2)	0.0162 (19)	0.0128 (19)	−0.0123 (19)
N1	0.045 (2)	0.047 (2)	0.058 (3)	0.0075 (18)	0.0063 (19)	0.0187 (19)
N2	0.042 (2)	0.042 (2)	0.056 (2)	0.0049 (16)	0.0049 (17)	0.0180 (17)
N3	0.046 (2)	0.049 (2)	0.0442 (19)	−0.0205 (17)	−0.0080 (16)	0.0169 (17)
N4	0.055 (2)	0.049 (2)	0.039 (2)	−0.0110 (18)	0.0065 (18)	0.0142 (17)
N5	0.050 (2)	0.044 (2)	0.045 (2)	−0.0143 (17)	0.0165 (17)	0.0115 (17)
N6	0.048 (2)	0.047 (2)	0.051 (2)	−0.0154 (17)	0.0111 (17)	−0.0098 (18)
O1	0.0475 (17)	0.0440 (18)	0.0475 (18)	0.0113 (13)	0.0030 (14)	0.0084 (15)
O2	0.0545 (19)	0.0475 (17)	0.0449 (17)	0.0085 (14)	−0.0040 (16)	0.0148 (16)
Co1	0.0466 (3)	0.0478 (3)	0.0446 (3)	−0.0004 (3)	−0.0022 (3)	0.0112 (3)
O3	0.0485 (16)	0.0416 (16)	0.0449 (16)	−0.0065 (13)	−0.0155 (15)	0.0075 (15)
O4	0.0540 (18)	0.0358 (16)	0.0512 (18)	−0.0083 (14)	0.0009 (15)	0.0047 (14)
O5	0.0458 (17)	0.0489 (19)	0.0522 (19)	0.0146 (13)	−0.0144 (14)	−0.0177 (15)
O6	0.0505 (17)	0.0525 (18)	0.0456 (18)	−0.0203 (15)	0.0137 (14)	−0.0169 (14)
O7	0.040 (3)	0.038 (3)	0.053 (4)	0.010 (2)	0.015 (3)	0.014 (3)

### Geometric parameters (Å, °)

**Table:**

C1—N1	1.335 (6)	C18—O4	1.268 (5)
C1—C2	1.381 (6)	C18—O3	1.275 (5)
C1—H1	0.9300	C19—N5	1.482 (6)
C2—C3	1.389 (6)	C19—H19A	0.9600
C2—H2	0.9300	C19—H19B	0.9600
C3—N2	1.399 (6)	C19—H19C	0.9600
C3—C4	1.401 (6)	C20—N5	1.478 (5)
C4—C5	1.367 (7)	C20—H20A	0.9600
C4—H4	0.9300	C20—H20B	0.9600
C5—N1	1.336 (6)	C20—H20C	0.9600
C5—H5	0.9300	C21—O5	1.238 (5)
C6—N4	1.329 (6)	C21—N5	1.449 (6)
C6—C7	1.338 (6)	C21—H21A	0.9300
C6—H6	0.9300	C22—N6	1.477 (6)
C7—C8	1.399 (6)	C22—H22A	0.9600
C7—H7	0.9300	C22—H22B	0.9600
C8—N3	1.404 (6)	C22—H22C	0.9600
C8—C9	1.405 (6)	C23—N6	1.486 (6)
C9—C10	1.315 (6)	C23—H23A	0.9600
C9—H9	0.9300	C23—H23B	0.9600
C10—N4	1.346 (6)	C23—H23C	0.9600
C10—H10	0.9300	C24—O6	1.276 (5)
C11—O2	1.242 (6)	C24—N6	1.465 (6)
C11—O1	1.246 (6)	C24—H24A	0.9300
C11—C12	1.522 (5)	N1—Co1	2.079 (4)
C12—C13	1.3900	N2—N3	1.316 (5)
C12—C17	1.3900	N4—Co1^i^	2.062 (4)
C13—C14	1.3900	O1—Co1	2.153 (3)
C13—H13	0.9300	O2—Co1	2.153 (3)
C14—C15	1.3900	Co1—O3^ii^	2.059 (3)
C14—H14	0.9300	Co1—N4^iii^	2.062 (4)
C15—C16	1.3900	Co1—O4^ii^	2.353 (3)
C15—C18	1.477 (5)	O3—Co1^iv^	2.059 (3)
C16—C17	1.3900	O4—Co1^iv^	2.353 (3)
C16—H16	0.9300	O7—H7A	0.8499
C17—H17	0.9300	O7—H7C	0.8501
			
N1—C1—C2	124.6 (4)	H19A—C19—H19C	109.5
N1—C1—H1	117.7	H19B—C19—H19C	109.5
C2—C1—H1	117.7	N5—C20—H20A	109.5
C1—C2—C3	118.8 (4)	N5—C20—H20B	109.5
C1—C2—H2	120.6	H20A—C20—H20B	109.5
C3—C2—H2	120.6	N5—C20—H20C	109.5
C2—C3—N2	119.9 (4)	H20A—C20—H20C	109.5
C2—C3—C4	117.1 (4)	H20B—C20—H20C	109.5
N2—C3—C4	123.0 (4)	O5—C21—N5	123.8 (4)
C5—C4—C3	119.0 (4)	O5—C21—H21A	118.1
C5—C4—H4	120.5	N5—C21—H21A	118.1
C3—C4—H4	120.5	N6—C22—H22A	109.5
N1—C5—C4	124.8 (4)	N6—C22—H22B	109.5
N1—C5—H5	117.6	H22A—C22—H22B	109.5
C4—C5—H5	117.6	N6—C22—H22C	109.5
N4—C6—C7	124.5 (4)	H22A—C22—H22C	109.5
N4—C6—H6	117.7	H22B—C22—H22C	109.5
C7—C6—H6	117.7	N6—C23—H23A	109.5
C6—C7—C8	119.9 (4)	N6—C23—H23B	109.5
C6—C7—H7	120.1	H23A—C23—H23B	109.5
C8—C7—H7	120.1	N6—C23—H23C	109.5
C7—C8—N3	122.8 (4)	H23A—C23—H23C	109.5
C7—C8—C9	115.9 (4)	H23B—C23—H23C	109.5
N3—C8—C9	121.3 (4)	O6—C24—N6	114.1 (4)
C10—C9—C8	118.7 (4)	O6—C24—H24A	122.9
C10—C9—H9	120.6	N6—C24—H24A	122.9
C8—C9—H9	120.6	C1—N1—C5	115.6 (4)
C9—C10—N4	126.3 (4)	C1—N1—Co1	117.3 (3)
C9—C10—H10	116.9	C5—N1—Co1	127.1 (3)
N4—C10—H10	116.9	N3—N2—C3	119.0 (4)
O2—C11—O1	122.7 (4)	N2—N3—C8	121.1 (3)
O2—C11—C12	117.6 (4)	C6—N4—C10	114.6 (4)
O1—C11—C12	119.6 (4)	C6—N4—Co1^i^	124.7 (3)
O2—C11—Co1	61.4 (2)	C10—N4—Co1^i^	120.7 (3)
O1—C11—Co1	61.4 (2)	C21—N5—C20	125.1 (4)
C12—C11—Co1	174.3 (3)	C21—N5—C19	119.8 (4)
C13—C12—C17	120.0	C20—N5—C19	115.0 (4)
C13—C12—C11	121.7 (2)	C24—N6—C22	119.6 (4)
C17—C12—C11	118.3 (2)	C24—N6—C23	120.7 (3)
C12—C13—C14	120.0	C22—N6—C23	118.1 (4)
C12—C13—H13	120.0	C11—O1—Co1	88.0 (3)
C14—C13—H13	120.0	C11—O2—Co1	88.2 (3)
C15—C14—C13	120.0	O3^ii^—Co1—N4^iii^	100.02 (14)
C15—C14—H14	120.0	O3^ii^—Co1—N1	95.89 (14)
C13—C14—H14	120.0	N4^iii^—Co1—N1	96.03 (14)
C16—C15—C14	120.0	O3^ii^—Co1—O2	156.86 (12)
C16—C15—C18	118.9 (2)	N4^iii^—Co1—O2	94.61 (14)
C14—C15—C18	121.0 (2)	N1—Co1—O2	100.31 (14)
C17—C16—C15	120.0	O3^ii^—Co1—O1	101.14 (13)
C17—C16—H16	120.0	N4^iii^—Co1—O1	154.34 (15)
C15—C16—H16	120.0	N1—Co1—O1	96.06 (14)
C16—C17—C12	120.0	O2—Co1—O1	60.96 (12)
C16—C17—H17	120.0	O3^ii^—Co1—O4^ii^	59.18 (11)
C12—C17—H17	120.0	N4^iii^—Co1—O4^ii^	92.07 (13)
O4—C18—O3	119.2 (4)	N1—Co1—O4^ii^	154.85 (14)
O4—C18—C15	120.9 (4)	O2—Co1—O4^ii^	102.73 (12)
O3—C18—C15	119.9 (3)	O1—Co1—O4^ii^	86.45 (11)
N5—C19—H19A	109.5	C18—O3—Co1^iv^	97.3 (2)
N5—C19—H19B	109.5	C18—O4—Co1^iv^	84.1 (3)
H19A—C19—H19B	109.5	H7A—O7—H7C	109.5
N5—C19—H19C	109.5		

Symmetry codes: (i) *x*, *y*, *z*−1; (ii) −*x*+3/4, *y*−1/4, *z*+1/4; (iii) *x*, *y*, *z*+1; (iv) −*x*+3/4, *y*+1/4, *z*−1/4.

### Hydrogen-bond geometry (Å, °)

**Table:**

*D*—H···*A*	*D*—H	H···*A*	*D*···*A*	*D*—H···*A*
O7—H7A···N5^ii^	0.85	2.32	2.932 (7)	129
C7—H7···O7	0.93	2.30	3.126 (7)	147
C16—H16···O3	0.93	2.48	2.791 (4)	100
C17—H17···O1	0.93	2.49	2.800 (4)	100
C19—H19C···O1^iv^	0.96	2.37	3.150 (6)	138
C23—H23A···O6^v^	0.96	1.71	2.624 (5)	158

Symmetry codes: (ii) −*x*+3/4, *y*−1/4, *z*+1/4; (iv) −*x*+3/4, *y*+1/4, *z*−1/4; (v) −*x*+1, −*y*+1/2, *z*+1/2.
